# Dynamical Basis for Drug Resistance of HIV-1 Protease

**DOI:** 10.1186/1472-6807-11-31

**Published:** 2011-07-08

**Authors:** Yi Mao

**Affiliations:** 1 National Institute for Mathematical and Biological Synthesis, University of Tennessee, Knoxville, TN 37996, USA

## Abstract

**Background:**

Protease inhibitors designed to bind to protease have become major anti-AIDS drugs. Unfortunately, the emergence of viral mutations severely limits the long-term efficiency of the inhibitors. The resistance mechanism of these diversely located mutations remains unclear.

**Results:**

Here I use an elastic network model to probe the connection between the global dynamics of HIV-1 protease and the structural distribution of drug-resistance mutations. The models for study are the crystal structures of unbounded and bound (with the substrate and nine FDA approved inhibitors) forms of HIV-1 protease. Coarse-grained modeling uncovers two groups that couple either with the active site or the flap. These two groups constitute a majority of the drug-resistance residues. In addition, the significance of residues is found to be correlated with their dynamical changes in binding and the results agree well with the complete mutagenesis experiment of HIV-1 protease.

**Conclusions:**

The dynamic study of HIV-1 protease elucidates the functional importance of common drug-resistance mutations and suggests a unifying mechanism for drug-resistance residues based on their dynamical properties. The results support the robustness of the elastic network model as a potential predictive tool for drug resistance.

## Background

HIV-1 protease (human immunodeficiency virus type 1 protease) is an enzyme that plays a critical role in the virus replication cycle. It cleaves the *gag *and *pol *viral polyproteins at the active site to process viral maturation [1-3], and without HIV-1 protease the virus was found to be noninfectious [4]. Thus HIV-1 protease is widely considered the major target for AIDS treatment [5,6]. One of the most severe obstacles to protease-inhibiting drugs is the rapid emergence of protease variants. Variants are able to evolve resistance by developing a chain of mutations, and as a result limit the long-term efficiency of these drugs [7,8].

HIV-1 protease is a dimer of C2 symmetry with each monomer consisting of 99 amino acid residues. Each monomer has one α helix and two antiparallel β sheets in the secondary structure. The enzyme active site is a catalytic triad composed of Asp25-Thr26-Gly27 from each monomer. It is gated by two extended β hairpin loops (residues 46−56) known as flaps [9]. At the molecular level, resistance to protease inhibition predominantly takes the form of mutations within the protein that preferentially lower the affinity of protease inhibitors with respect to protease substrates, while still maintaining a viable catalytic activity [10]. Mutations associated with drug resistance occur within the active site as well as non-active distal sites [11].

During the past two decades, researchers and clinicians from different disciplines have made enormous efforts to investigate resistance against HIV-1 protease targeted drugs. To elucidate the molecular mechanisms of drug resistance, biochemists and molecular biologists have characterized the structure, energetics and catalytic efficiency of a large number of HIV-1 protease mutants to unravel the resistance mechanism in combination with extensive computational studies [12-15]. Moreover, drug resistance data collected from AIDS patients treated with HIV-1 protease inhibitor drugs [16-19] provide opportunities for researchers to identify resistance-related mutation patterns [20-22]. Recently there have been efforts to link protein physical and functional stability with its evolutionary dynamics [23,24].

At the heart of understanding the molecular basis of drug-resistant behaviors of HIV-1 protease is the structural distribution of resistance mutations. Presumably these mutations are not randomly located throughout the protein structure. Although different HIV-1 protease inhibitors elicit different combinations of mutation types to generate distinctive resistance levels, there are 21 most common mutations associated with resistance against all inhibitors [19]. Prediction of resistance mutations of proteins is based on either sequence or structure information [25]. Sequence-based methods predict resistance mutations by analyzing large datasets of sequences with known resistance properties. Thus the availability of those datasets is a prerequisite for such methods [22,26-28]. On the other hand, predicting mutations using protein structure has largely relied on the characterization of binding thermodynamics [29-32], as the mutations with resistance against inhibitors lower the binding affinity of inhibitors far more than that of natural substrates. The accuracy of the prediction is directly related to the accuracy of the potential function used in the calculations and the adequacy of the sampling of the protein conformational space. It is also sensitive to the error/noise in the free energy calculations [32].

Conformational dynamics play an essential role in regulating protein function [33,34]. In the past few years a deepening understanding of the relationship of protein dynamics and function has emerged [35]. Relevant to the study here is the utilization of protein dynamics to identify the sequence regions of functional importance even though their locations may be remote from the active site. Computationally there have been rapid methodological developments in relating protein dynamics to function by probing the long range communications between residues: perturbation method [36,37], clustering analysis of correlation matrix [38], network analysis [39], and energy diffusivity estimation by propagation through vibrational modes [40]. The success of these methods in reproducing experimental results as well as findings from sequence-based methods has established the validity of dynamics-based approaches [38,41].

The dynamics of HIV-1 protease, especially binding dynamics of its ligands are fundamental to the protease inhibitor design and have been a subject of intense computational study [42-49]. Because of limitations of time scale in all-atom simulations, various coarse-grained models have been used to investigate HIV-1 protease binding dynamics and kinetics, shedding light on important dynamics issues [45-49]. The main features of substrate interactions and dynamics at the active site were analyzed within the framework of the coarse-grained model [45,49]. Gaussian models were shown to describe accurately the correlated motion of HIV-1 protease residues in thermodynamic equilibrium through a series of successful comparisons with an extensive MD simulation [49,50]. There is increasing evidence relating protease's drug-resistance mutations to its dynamics. The impact of some distal mutations on catalytic function of HIV-1 protease was linked to protein flexibility [51,52]. Multi-drug resistance residues of HIV-1 protease were found to overlap the global hinge region identified from coarse-grained normal-mode analysis of the protease [53]. Nevertheless, despite extensive research efforts, a general explanation for drug-resistance mutations of HIV-1 protease is still lacking [54].

In this study, a coarse-grained elastic network model is used to investigate the dynamics of HIV-1 protease, to probe the connection between its global dynamics and the distribution of drug-resistance mutations, and to examine the potential of the dynamics-based approach as a predictive tool for drug resistance prediction, with an attempt to provide a unifying mechanistic explanation for all residues of resistance based on their dynamical properties. The crystal structures of an unbound form and bound forms with a substrate and nine FDA approved inhibitors of HIV-1 protease are used as model systems. Correlation analysis of the protease at equilibrium focuses on two functional sites of HIV-1 protease: the active site (the Asp25-Thr26-Gly27 triad [45]) and the flap (residues 45-55 [47]). The protease dynamic changes upon ligand binding are examined as well. The implications for resistance mechanisms and protein evolution are discussed.

## Results

Here HIV-1 protease is represented by a coarse-grained network model, and its dynamics is examined in several X-ray crystallographic structures. The linkage between global dynamics and the distribution of drug-resistance mutations is examined first in individual unbound and bound forms, then in the dynamical differences between the unbound and bound forms. The former is a measure of the residual fluctuations in different structures, and the latter is an estimate of dynamical change caused by ligand binding.

### Dynamically coupled regions identified by equilibrium correlations Unbound form

**Figure 1:**
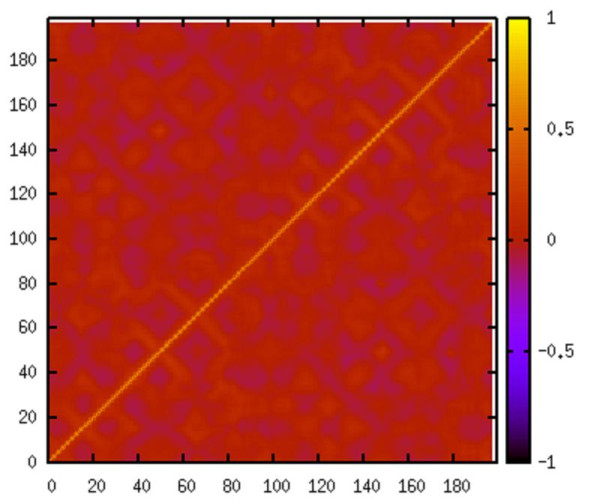
**Correlation matrix**. The correlation matrix for an unbound form of HIV-1 protease (PDB id 1HHP).

**Figure 2:**
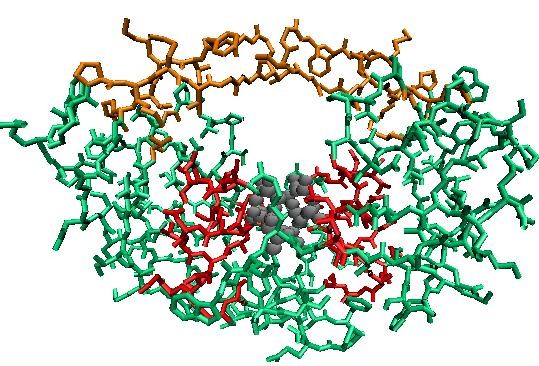
**Two clusters containing functional sites**. Residues in Clusters 4 (red) and 5 (yellow) are displayed in an unbound form of HIV-1 protease (PDB id 1HHP). The active site is highlighted by a ball representation.

The correlation matrix, consisting of correlations of all residue pairs, captures the essence of protein dynamics. A 198 × 198 correlation matrix (Figure 1) is generated from the elastic network modeling (see Methods section) based on the unbound HIV-1 protease (PDB id 1HHP). The most conspicuous features in the figure are the beta-sheets, represented by the line across the diagonal (residues 19-24, 43-66, 69-78, 118-123, 142-165 and 168-177). Extraction of further information from the matrix requires the application of data analysis tools such as clustering algorithms. The Markov cluster (MCL) algorithm (see Methods section) is the chosen method, and the results from the MCL program for the unbound form consist of five clusters (Table 1). Cluster 1 is the largest cluster with 48 residues. It covers the core domain, which excludes both termini and the flap, and forms the scaffold surrounding the active site of the enzyme. Clusters 2 and 3 are exclusively composed of the residues located at the beginning of the N-terminus and the end of the C-terminus, respectively. The remaining two clusters concentrate on two functional sites (Figure 2). Cluster 4 contains the active site of HIV-1 protease (Asp25-Thr26-Gly27 catalytic triad), one residue at the dimerization region (Leu10), and a large segment of the C-terminal (Ile84-Cys95). Cluster 5 is mainly made up of the residues at the flap region (Pro44-Arg57) with two additional residues from the C-terminal domain (Leu76 and Gly78). These two functional sites are involved in distinct aspects of the protease function: the active site is where the enzymatic catalysis takes place, and the flap controls access to the active site. The clustering results indicate that these two functional sites belong to separate interaction networks of the protease.

**Table 1:** Clustering result of the unbound form

Scaffold	11 12 13 14 15 16 17 18 19 20 21 22 32 33 34 35 36 37 38 39 40 41 42 43 58 59 60 61 62 63 64 65 66 67 68 69 70 71 72 73 74 75 77 79 80 81 82 83
N-terminal	1 2 3 4 5 6 7 8 9
C-terminal	96 97 98 99
Coupled with the active site	10 23 24 25 26 27 28 29 30 31 84 85 86 87 88 89 90 91 92 93 94 95
Coupled with the flap region	44 45 46 47 48 49 50 51 52 53 54 55 56 57 76 78

Residues of the active site and of the flap are underlined and double-underlined, respectively.

### Bound forms

The ten structures of HIV-1 protease in complex with the natural substrate and nine FDA approved inhibitors are chosen to represent HIV-1 protease in bound forms. Despite their varying degrees of similarity with the unbound form, these ten structures generate very similar clustering results (Table 2). The general clustering pattern of HIV-1 protease unbound form is preserved in all the bound forms: the scaffold, the N- and C- termini, the active site and the flap. Nevertheless, inside the clusters there are reorganizations and splits of the clusters due to ligand binding. Upon binding, the tips of the flaps (residues 48-55) close and cover the active site, and the physical proximity facilitates stronger interactions between the flap region and part of the C-terminal. As a result, the cluster containing the flap grows in the bound form. The cluster containing the flap is enlarged by including residues 79-83, while part of the C-terminal (residues 87-95) is disengaged from the cluster with the active site and forms a new cluster of its own. The diversity of the ligand types of the ten structures does not induce a dramatic impact on the clusters. The number of residues in the cluster of the active site and of the flap ranges from 12 to 18 and from 19 to 24 respectively, but the number of separate sequence segments in these two clusters remains constant.

**Table 2:** Clustering results of the ten bound forms

PDB id	Mutation	RMSD (Å)	Coupled with the active site	Coupled with the flap region
2FNS 7 (substrate)	Q7K, D25N, L63P, I64V	0.52	10*23 24 25 26 27 28 29 *30 31 84*85 86 *87	*44 45 464748 49 50 51 52 5354 55 56 *78 *79 80 81 82*83
1HPV 6 (Amprenavir)	None	0.54	10*23 24 25 26 27 28 29 *30 31 32*85 86*	*44 45 464748 49 50 51 52 5354 55 56 *78 *79 80 81 82*83 84
1HXB 6 (Saquinavir)	None	0.31	10*23 24 25 26 27 28 29 *30 31 32*85 86*	*44 45 464748 49 50 51 52 5354 55 56 *78 *79 80 81 82*83 84
1MUI 5 (Lopinavir)	N37S	0.53	10*23 24 25 26 27 28 29 *30 31 3284*85 86*	*44 45 464748 49 50 51 52 5354 55 56 *78 *79 80 81 82*83
2O4K 8 (Atazanavir)	Q7K	0.35	*23 24 25 26 27 28 29 *30 31 32 83 84*85 86 *87 88 89 90	43 *44 45 464748 49 50 51 52 5354 55 56 *57 58 7677 78 *79 80 81 82*
3JVY 6 (Darunavir)	Q7K, L33I*, L63I, C67A, G86A, C95A	0.38	*23 24 25 26 27 28 29 *30 31 32*85 86*	*44 45 464748 49 50 51 52 5354 55 56 79 80 81 82*83 84
2O4P 8 (Tipranavir)	Q7K	0.39	*23 24 25 26 27 28 29 *30 31 32 83 84*85 86 *87 88 89 90	*44 45 464748 49 50 51 52 5354 55 56 79 80 81 82*83 84
1HXW 6 (Ritonavir)	None	0.40	10*23 2425 26 27 28 29 *30 31 *85 86 *87	*44 45 464748 49 50 51 52 5354 55 56 79 80 81 82*83 84
2PYN 6 (Nelfinavir)	D30N, A71V*	0.26	10*23 24 25 26 27 28 29 *30 31 3284*85 86*	*44 45 464748 49 50 51 52 5354 55 56 *78 *79 80 81 82*83
2B7Z 7 (Indinavir)	K20R*, V32I*, L33F*, M36I*, M46I*, L63P, A71V*,V82A*, I84V*, L90M*	0.32	10 22 *23 24 25 26 27 28 29 *84*85 86*	34 43 *44 45 464748 49 50 51 52 5354 55 56 *57 7677 78 *79 80 81 82*83

The type of the ligand is listed in brackets. The global average RMSD of backbone atoms between the unbound (PDB id 1HHP) and bound forms is given. The mutations of each protease are listed. Only the clusters containing the active site and the flap are shown here.

(1) *drug-resistance mutations

(2) Residues belonging to the 21 common resistant mutations are underlined.

(3) Residues shared by all the bound forms are in italics.

In summary, there exist two independent clusters containing two important functional sites of HIV-1 protease. These two networks are relatively robust to perturbations caused by the different types of ligand.

### Dynamical behavior differences between the unbound and bound forms

**Figure 3:**
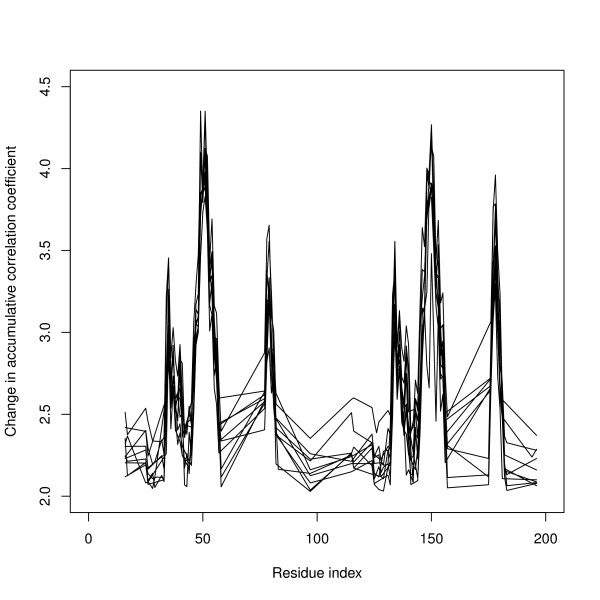
**Fluctuation differences between the unbound and bound forms**. Fluctuation difference between the unbound (PDB id 1HHP) and ten bound forms of HIV-1 protease plotted as a function of residues. Only residues with larger than average difference are plotted.

Without directly engaging the active site, another way to influence a protein's function is to perturb the motions essential to its function. For enzymes, these essential motions are the conformational changes accompanied by the association and dissociation of ligands [55]. In HIV-1 protease the large scale open/close conformational change of the flap along the reaction pathway is the major structural reorganization induced by ligand binding [43-47]. Extensive investigations of 73 X-ray mutant and complex structures of HIV-1 protease revealed that a common and predominant dynamic behavior was found among the protease in complex with different ligands [56]. The focus of study here is the dynamical changes from the unbound to bound forms of HIV-1 protease at the residue level. The dynamical property of each residue is characterized by the sum of its couplings with other residues (see Methods). The binding-induced changes in the dynamical behaviors of individual residues were very similar among the ten bound structures (Figure 3). Overall, the perturbations to the residues are not isotropic, and the regions exhibiting the largest deviations are signaled by peaks. The ten bound proteases all share very similar locations and magnitudes of these peaks (residues 32-42, 44-57 and 77-82). The most pronounced difference between the unbound and bound protease is the loss of flexibility in the flap tip upon binding, as indicated by the highest peak (around residues 44-57) in Figure 3.

## Discussion

Using a coarse-grained network model, functionally important residues of HIV-1 protease were identified based on correlation analysis of either equilibrium fluctuations or dynamical changes. Experimentally the residues of functional importance can be directly probed by mutagenesis experiments. The complete mutagenesis experiments carried out on HIV-1 protease revealed three mutationally sensitive sequence domains [57]: the active site region (Ala22-Leu33), the flap region (Ile47-Gly52) and the part of C-terminal (Thr74-Arg87). The active site and flap region have obvious functional significance. The influence the C-terminal residues exert on the catalytic cycle is most likely through long-range couplings with the functional sites. Correlation analysis suggests that some residues of the C-terminal (Ile84-Cys95) are functionally important due to their strong interactions with the active site, while the others (Leu76 and Gly78) may influence the dynamics of the protein through their couplings with the flap. The dynamical changes analysis also indicates that residues 77-82 are involved in the binding process. Structurally, residues 79-84 form a wall of the active site, and their motions were shown by previous simulations to correlate well with the open/closed dynamics of the flap [52].

Currently there are nine FDA approved protease inhibitors, and the most up-to-date clinical data indicate 21 most common drug-resistant mutation positions ("most common" defined as mutation position shared by at least two inhibitors among the nine currently approved protease inhibitors):10, 20, 24, 32, 33, 36, 46, 47, 48, 50, 53, 54, 62, 71, 73, 76, 77, 82, 84, 88 and 90 [19]. These mutations are located at the dimer interface (residues 88 and 90), the core domain (residues 10, 20, 24, 32, 33, 36, 62, 71, 73, 76, 77, 82, and 84), and the flap domain (residues 46, 47, 48, 50, 53, and 54), respectively. The clustering analysis of HIV-1 protease in various forms identified 10, 24, 32, 46, 47, 48, 50, 53, 54, 76, 77, 82, 84, 88 and 90 (15 out of the total 21 resistance sites) as those coupled either with the active site or the flap. The dynamical change analysis identifies two additional residues 33 and 36 as residues of importance. The undetected residues (20, 62, 71 and 73) are located in the hydrophobic core of the protease, and their mutations likely affect the protease activity through influencing the protease structure and stability [30]. Another noteworthy fact is that there are far more residues discovered by clustering analysis than by clinical studies (15 out of the total 34 residues in the two clusters containing functional sites are the residues of resistance). Drug resistance residues not only influence protease binding, but also generate differential binding affinity between the substrate and inhibitors. The cluster analysis can only detect residues that may influence the binding. Therefore the pool of residues identified by the clustering analysis is larger than those found with drug resistance affinity in clinical studies. The similar clustering results from different protease inhibitor complexes further suggest that global dynamics are preserved among different complexes of HIV-1 protease inhibitors. The convergence of results regardless of the conformation of the protein was also found by the Gaussian network modeling of HIV-1 protease [50]. Computational studies using atomistic MD simulations reached the same conclusion that correlation matrix based analysis does not differentiate the essential modes of motion of the protein native forms from those of the mutants [58]. Nevertheless, the dynamic approach proposed here locates a majority of drug-resistance mutations, and provides insight on the drug-resistance mechanism of HIV-1 protease. Drug resistance mutations of HIV-1 protease can be classified as active site or non-active site mutations, depending on their location within the protein. Active site mutations are located in the vicinity of the active site and directly affect the protease-inhibitor interactions. Thus their action on inhibitor binding affinity can be readily understood in structural terms. On the other hand, non-active site mutations influence binding from various distal locations and their mechanism of action is not immediately apparent. Although some residue-specific explanations and suggestions have been proposed, the overall mechanism by which these diversely located non-active site residues influence inhibitor-binding remains unclear [19]. Dynamic studies presented here suggest a simple yet general explanation for the distribution of the drug resistance residues in HIV-1 protease. Except for these residues of resistance influencing protease binding affinity by acting on the structural stability of HIV-1 protease, the drug-resistance residues belong to clusters that are either the "coupled with the active site" or "coupled with the flap". It is noteworthy that residues coupled with the functional sites (Clusters 4 and 5) do not all locate in the physical vicinity of each other. Long distance communications play an important role in mediating the interactions between the functional sites and distal residues. The residues clustered with the functional sites can exert an impact on protein function, even though they may not locate near the active site. Leu10 and Leu90, among the residues clustered with the active site (Cluster 3), are well-known residues whose mutations lead to drug resistant variants [17]. The finding that Leu76 and Gly78 are coupled with the flap region is also corroborated by a detailed protein flexibility study of HIV-1 protease which concluded that mutations at residues Thr74-Val77, although far from the active site, reduce protease activity because of their correlated motions with the flap region [55]. Traditionally, computer methods of predicting resistance mutations based on protein structure have been largely focused on energetic analysis [25], in which atomistic molecular mechanics and/or molecular dynamics are used to investigate ligand-protein binding affinity. The mechanism of non-active site mutations has largely remained a challenge for energetic analysis because of the minimal structural and energetic perturbations caused by those mutations [51]. These difficulties, however, open the door for dynamics-based study, especially via coarse-grained methods such as elastic network modeling, which provide an efficient way of sampling the global dynamics of proteins. The interaction network identified by the correlation analysis contains both active site and non-active site residues. These residues influence the inhibitor binding by coupling with the active site and the flap, regardless of their physical proximity. Thus the dynamic approach in this study is able to detect both active and non-active drug-resistance sites based on coarse-grained protein models.

It is believed that only a few amino acid sites are responsible for adaptive evolution in almost all proteins [59], although the nature of these positively selected residues is yet to be elucidated. The findings from this study indicate that the interaction networks of globally distributed residues involving the functional sites play a dominant role in the evolutionary pathways of HIV-1 protease, and they become the major sites that develop resistance under selective antiviral pressure. Pathogenic proteins such as HIV-1 protease escape the challenges to their survival imposed by drug inhibition through mutations at these amino acid residues. Structure-based modeling of proteins confirms the decisive role of physical interactions in the evolution of virus proteins and raises the possibility of constructing a protein fitness landscape by means of physical modeling of proteins.

## Conclusions

This study examines the functional significance of common drug-resistance mutations of HIV-1 protease by characterizing its global dynamics using coarse-grained modeling. The calculations show that most residues of drug-resistance are coupled either with the active site or with the flap. These couplings are rather robust to the perturbation of ligand binding. These findings result in a unifying mechanism for all drug-resistance residues based on their dynamical properties. They also indicate that global dynamics of HIV-1 protease are intrinsically connected to the structural distribution of drug-resistance mutations, thus dynamic study provides a simple yet general and useful tool to examine the tendency of drug resistance of residues in addition to traditional energetic analysis.

## Methods

### Elastic network modelling

The elastic network model was applied according to the standard protocol [60]. The details of the correlation matrix can be found elsewhere [41]. In this study the cutoff distance *R_c _*is set to be 10Å, but the choice of *R_c _*was shown not to noticeably affect the results based on the correlation matrix generated from the model [61]. The structures of the unbound and ligand-bound forms of HIV-1 protease with various ligands were used. For the ligand-bound protein, only the C*_α _*atoms of the protein are represented by the network model and the ligand is not incorporated in the model. The online server http://ignmtest.ccbb.pitt.edu/cgi-bin/anm/anm1.cgi[60] was used to generate the correlation matrix, and the element in the correlation matrix is defined as(1)

where Δ*R_i _*and Δ*R_j _*are the fluctuations of nodes *i *and *j*, respectively.

### Clustering analysis

The correlation matrix is submitted for clustering analysis by the Markov cluster (MCL) algorithm [62]. The MCL algorithm is one of the most successful clustering procedures in identifying protein-protein interactions from genomic data [63] and has been shown to be robust and outperform other clustering algorithms [64]. Relevant to the study here is the application of MCL to clustering protein residues based on the interaction correlation matrix [38]. MCL finds cluster structure in graphs by performing a random walk through the graphs. The process computes the probabilities of random walks through the graph, and uses expansion and inflation to change the probabilities associated with the random walks departing from one particular node. It results in the separation of the graph into different segments. Cluster granularity is controlled by the inflation parameter which is the only variable in the MCL program used in this study. In order to reduce the noise, the correlation matrix has to be adjusted before being submitted to the MCL program. First the absolute values of the correlation coefficients are taken. Then a cutoff value is applied to produce a condensed version. Correlations less than the cutoff value are set to zero and the cutoff value is subtracted from the remaining correlations. The cutoff and the inflation parameter (0.08 and 1.5, 0.075 and 1.4 for the unbound and bound protease, respectively) were chosen to produce a total number of five clusters for the unbound protease, and six to seven clusters for the bound protease. The number of clusters is chosen to be five or six because the resulting clusters make the most physical sense. All the bound forms are subject to the same parameters.

### Fluctuation difference between unbound and bound forms

The structural differences between the unbound (ligand-free) and bound (ligand-bound) proteins are usually significant. The structural changes are not to be identified with the dynamical behavior changes. The change in dynamical behavior caused by binding for residue *i*, Δ*C_i_*, is calculated as the sum of the absolute values of the difference between the correlation coefficients of residue *i *in the unbound and bound forms,(2)

In Equation 2, the sum is over all the residues in the protein. *C_ij _*and *C'_ij _*denote the correlation coefficient of residues *i *and *j *in the unbound and bound forms, respectively.

## Authors' contributions

YM is responsible for all the work related to the manuscript.

## References

[B1] Katz RA The retroviral enzymes Annu Rev Biochem 1994 63 133 173 10.1146/annurev.bi.63.070194.001025 7526778

[B2] Wlodawer A Rational approach to AIDS drug design through structural biology Ann Red Med 2002 53 595 614 10.1146/annurev.med.53.052901.131947 11818491

[B3] Kurup A Mekapati SB Garg R Hansch C HIV-1 protease inhibitors: a comparative QSAR analysis Curr Med Chem 2003 10 1679 1688 10.2174/0929867033457070 12871116

[B4] Kohl NE Emini EA Schleif WA Davis LJ Heimbach JC Dixon RA Scolnick EM Sigal IS Active human immunodeficiency virus protease is required for viral infectivity Proc Natl Acad Sci USA 1988 85 4686 4690 10.1073/pnas.85.13.4686 3290901 PMC280500

[B5] Wlodawer A Vondrasek J Inhibitors of HIV-1 protease: A major success of structureassisted drug design Annu Rev Biophys Biomol Struct 1998 27 249 284 10.1146/annurev.biophys.27.1.249 9646869

[B6] Blair WS HIV-1 entry- an expanding portal for drug discovery Drug Discovery Today 2000 5 183 194 10.1016/S1359-6446(00)01484-7 10790262

[B7] Condra JH Schleif WA Blahy OM Gabryelski LJ Graham DJ Quintero JC Rhodes A Robbins HL Roth E Shivaprakash M Titus D Yang T Tepplert H Squires KE Deutsch PJ EMini EA In-vivo emergence of hiv-1 variants resistant to multiple protease inhibitors Nature 1995 374 569 571 10.1038/374569a0 7700387

[B8] Ho DD Toyoshima T Mo H Kempf DJ Norbeck D Chen CM Wideburg NE Burt SK Erickson JW Singh MK Characterization of human immunodeficiency virus type 1 variants with increased resistant to a C2-symmetric protease inhibitor J Virol 1994 68 2016 2020 8107264 10.1128/jvi.68.3.2016-2020.1994 PMC236669

[B9] Mager PP The active site of HIV-1 protease Med Res Rev 2001 21 348 353 10.1002/med.1012 11410934

[B10] Freire E Overcoming HIV-1 resistance to protease inhibitors Drug Discov Today 2006 3 281 286 10.1016/j.ddmec.2006.06.005

[B11] Muzammil S Ross P Freire E A major role for a set of non-active site mutations in the development of HIV-1 protease drug resistance Biochemistry 2003 42 631 638 10.1021/bi027019u 12534275

[B12] Chen XF Weber IT Harrison RW Molecular dynamics simulations of 14 HIV protease mutants in complexes with indinavir J Mol Model 2004 10 373 381 10.1007/s00894-004-0205-x 15597206

[B13] Hou TJ McLaughlin WA Wang W Evaluating the potency of HIV-1 protease drugs to combat resistance Proteins 2008 71 1163 1174 18004760 10.1002/prot.21808 PMC2628484

[B14] Muzammil S Armstrong AA Kang LW Jakalian A Bonneau PR Schmelmer V Amzel LM Freire E Unique thermodynamic response of tipranavir to human immunodeficiency virus type 1 protease drug resistance mutations J Virol 2007 81 5144 5154 10.1128/JVI.02706-06 17360759 PMC1900215

[B15] Prabu-Jeyabalan M Nalivaika E Schiffer CA Substrate shape determines specificity of recognition for HIV-1 protease: analysis of crystal structures of six substrate complexes Structure 2002 10 369 381 10.1016/S0969-2126(02)00720-7 12005435

[B16] Hertogs K Bloor S Kemp SD Van den Eynde C Alcorn TM Pauwels R Houtte MV Staszewski S Miller V Larder BA Phenotypic and genotypic analysis of clinical HIV-1 isolates reveals extensive protease inhibitor cross-resistance: a survey of over 6000 samples AIDS 2000 14 1203 1210 10.1097/00002030-200006160-00018 10894285

[B17] Shafer RW Genotypic testing for human immunodeficiency virus type 1 drug resistance Clin Microbiol Rev 2002 15 247 277 10.1128/CMR.15.2.247-277.2002 11932232 PMC118066

[B18] Chen L Perlina A Lee CJ Positive selection detection in 40,000 human immunodeficiency virus (HIV) type 1 sequences automatically identifies drug resistance and positive fitness mutations in HIV protease and reverse transcriptase J Virol 2004 78 3722 3732 10.1128/JVI.78.7.3722-3732.2004 15016892 PMC371046

[B19] Johnson VA Brun-Vezinet F Clotet B Gunthard HF Kuritzkes DR Pillay D Schapiro JM Richman DD Update of the drug resistance mutations in HIV-1: December 2009 Top HIV Med 2009 17 138 145 20068260

[B20] Vercauteren J Vandamme AM Algorithms for the interpretation of HIV-1 genotypic drug resistance information Antivir Res 2006 71 335 342 10.1016/j.antiviral.2006.05.003 16782210

[B21] Beerenwinkel N Schmidt B Walter H Kaiser R Lengauer T Hoffmann D Korn K Selbig J Diversity and complexity of HIV-1 drug resistance: a bioinformatics approach to predicting phenotype from genotype Proc Natl Acad Sci USA 2002 99 8271 8276 10.1073/pnas.112177799 12060770 PMC123057

[B22] Rhee SY Taylor J Wadhera G Ben-Hur A Brutlag DL Shafer RW Genotypic predictors of human immunodeficiency virus type 1 drug resistance Proc Natl Acad Sci USA 2006 103 17355 17360 10.1073/pnas.0607274103 17065321 PMC1622926

[B23] Hamacher K Relating sequence evolution of HIV1-protease to its underlying molecular mechanics Gene 2008 422 30 36 10.1016/j.gene.2008.06.007 18590806

[B24] Zhang J Hou T Wang W Liu JS Detecting and understanding combinatorial mutation patterns responsible for HIV drug resistance Proc Natl Acad Sci USA 2010 107 1321 1326 10.1073/pnas.0907304107 20080674 PMC2824344

[B25] Cao ZW Han LY Zheng CJ Ji ZL Chen X Lin HH Chen YZ Computer prediction of drug resistance mutations in proteins Drug Discov Today 2005 10 521 529 10.1016/S1359-6446(05)03377-5 15809198

[B26] Saigo H Uno T Tsuda K Mining complex genotypic features for predicting HIV-1 drug resistance Bioinformatics 2007 23 2455 2462 10.1093/bioinformatics/btm353 17698858

[B27] Wang K Jenwitheesuk E Samudrala R Mittler JE Simple linear model provides highly accurate genotypic predictions of HIV-1 drug resistance Antivir Ther 2004 9 343 352 15259897

[B28] Zazzi M Romano L Venturi G Shafer RW Reid C Dal Bello F Parolin C Palù G Valensin PE Comparative evaluation of three computerized algorithms for prediction of antiretroviral susceptibility from HIV type 1 genotype J Antimicrob Chemother 2004 53 356 360 10.1093/jac/dkh021 14688053

[B29] Wang W Kollman PA Computational study of protein specificity: the molecular basis of HIV-1 protease drug resistance Proc Natl Acad Sci USA 2001 98 14937 14942 10.1073/pnas.251265598 11752442 PMC64962

[B30] Chen YZ Gu XL Cao ZW Can an optimization/scoring procedure in ligand protein docking be employed to probe drug resistant mutations in proteins? J Mol Graph Model 2001 19 560 570 10.1016/S1093-3263(01)00091-2 11552685

[B31] Shenderovich MD Kagan RM Heseltine PNR Ramnarayan K Structure-based phenotyping predicts HIV-1 protease inhibitor resistance Protein Sci 2003 12 1706 1718 10.1110/ps.0301103 12876320 PMC2323957

[B32] Hou T Zhang W Wang J Wang W Predicting drug resistance of the HIV-1 protease using molecular interaction energy components Proteins 2009 74 837 846 10.1002/prot.22192 18704937 PMC3210569

[B33] Eisenmesser EZ Millet O Labeikovsky W Korzhnev DM Wolf-Watz M Bosco DA Skalicky JJ Kay LE Kern D Intrinsic dynamics of an enzyme underlies catalysis Nature 2005 438 117 121 10.1038/nature04105 16267559

[B34] Kern D Zuiderweg ERP The role of dynamics in allosteric regulation Nature 2003 13 748 757 10.1016/j.sbi.2003.10.008 14675554

[B35] Bahar I Lezon TR Yang LW Eyal E Global dynamics of proteins: bridging between structure and function Annu Rev Biophys 2010 39 23 42 10.1146/annurev.biophys.093008.131258 20192781 PMC2938190

[B36] Zheng W Tekpinar M Large-scale evaluation of dynamically important residues in proteins predicted by the perturbation analysis of a coarse-grained elastic model BMC Struct Biol 2009 9 45 10.1186/1472-6807-9-45 19591676 PMC2719638

[B37] Sharp K Skinner JJ Pump-probe molecular dynamics as a tool for studying protein motion and long range coupling Proteins 2006 65 347 361 10.1002/prot.21146 16933296

[B38] Kong Y Karplus M Signaling pathways of PDZ2 domain: A molecular dynamics interaction correlation analysis Proteins 2009 74 145 154 10.1002/prot.22139 18618698 PMC2605193

[B39] Cusack MP Thibert B Bredesen DE del Rio G Efficient identification of critical residues based only on protein structure by network analysis PLoS ONE 2007 2 e421 10.1371/journal.pone.0000421 17502913 PMC1855080

[B40] Leitner DM Frequency-resolved communication maps for proteins and other nanoscale materials J Chem Phys 2009 130 195101 195109 10.1063/1.3130149 19466865

[B41] Mao Y Dynamics studies of luciferase using elastic network model: how the sequence distribution of luciferase determines its color Protein Eng Des Sel 2011 24 341 349 10.1093/protein/gzq109 21159621

[B42] Song Y Zhang Y Shen T Bajaj CL McCammon JA Baker NA Finite element solution of the steady-state Smoluchowski equation for rate constant calculations Biophys J 2004 86 2017 2029 10.1016/S0006-3495(04)74263-0 15041644 PMC1304055

[B43] Hornak V Okur A Rizzo RC Simmerling C HIV-1 protease flaps spontaneously close to the correct structure in simulations following manual placement of an inhibitor into the open state J Am Chem Soc 2006 128 2812 2813 10.1021/ja058211x 16506755 PMC2555982

[B44] Toth G Borics A Closing of the flaps of HIV-1 protease induced by substrate binding: A model of flap closing mechanism in retroviral aspartic proteases Biochemistry 2006 45 6606 6614 10.1021/bi060188k 16716071

[B45] Li D Liu MS Ji B Hwang K Coarse-grained molecular dynamics of ligands binding into protein: the case of HIV-1 protease inhibitors J Chem Phys 2009 130 215102 10.1063/1.3148022 19508101

[B46] Tozzini V McCammon JA A coarse grained model for the dynamics of flap opening in HIV-1 protease Chem Phys Lett 2005 413 123 128 10.1016/j.cplett.2005.07.075

[B47] Tozzini V Trylska J Chang C McCammon JA Flap opening dynamics in HIV-1 protease explored with a coarse-grained model J Struct Biol 2007 157 606 615 10.1016/j.jsb.2006.08.005 17029846

[B48] Pandey RB Farmer BL Residue energy and mobility in sequence to global structure and dynamics of a HIV-1 protease (1DIFA) by a coarse-grained Monte Carlo simulation J Chem Phys 2009 130 044906 10.1063/1.3050106 19191412

[B49] Micheletti C Carloni P Maritan A Accurate and efficient description of protein vibrational dynamics: comparing molecular dynamics and Gaussian models Proteins 2004 55 635 645 10.1002/prot.20049 15103627

[B50] Kurt N Scott WR Schiffer CA Haliloglu T Cooperative fluctuations of unliganded and substrate-bound HIV-1 protease: A structure-based analysis on a variety of conformations from crystallography and molecular dynamics simulations Proteins 2003 51 409 422 10.1002/prot.10350 12696052

[B51] Piana S Carloni P Rothlisberger U Drug resistance in HIV-1 protease: Flexibility assisted mechanism of compensatory mutations Protein Sci 2002 11 2393 2402 12237461 10.1110/ps.0206702 PMC2384161

[B52] Perryman AL Lin JH McCammon JA HIV-1 protease molecular dynamics of a wild-type and of the V82F/I84V mutant: possible contributions to drug resistance and a potential new target site for drugs Protein Sci 2004 13 1108 1123 10.1110/ps.03468904 15044738 PMC2280056

[B53] Liu Y Eyal E Bahar I Analysis of correlated mutations in hiv-1 protease using spectral clustering Bioinformatics 2008 24 1243 1250 10.1093/bioinformatics/btn110 18375964 PMC2373918

[B54] Ali A Bandaranayake RM Cai Y King NM Kolli M Mittal S Murzycki JF Nalam MNL Nalivaika EA Özen A Prabu-Jeyabalan MM Thayer K Schiffer CA Molecular basis for drug resistance in HIV-1 protease Viruses 2010 2 2509 2535 10.3390/v2112509 PMC3185577 21994628

[B55] Szarecka A Xu Y Tang P Dynamics of firefly luciferase inhibition by general anesthetics: Gaussian and anisotropic network analyses Biophys J 2007 93 1895 1905 10.1529/biophysj.106.102780 17513367 PMC1959537

[B56] Zoete V Michielin O Karplus M Relation between sequence and structure of HIV-1 protease inhibitor complexes: a model system for the analysis of protein flexibility J Mol Biol 2002 315 21 52 10.1006/jmbi.2001.5173 11771964

[B57] Loeb DD Swanstrom R Everitt L Manchester M Stamper SE Hutchison CA III Complete mutagenesis of the HIV-1 protease Nature 1989 340 397 340 10.1038/340397a0 2666861

[B58] Genoni A Morra G Merz KM Jr Colombo G Computational study of the resistance shown by the subtype B/HIV-1 protease to currently known inhibitors Biochemistry 2010 49 4283 4295 10.1021/bi100569u 20415450 PMC2868114

[B59] Guindon S Rodrigo AG Dyer KA Huelsenbeck JP Modeling the site-specific variation of selection patterns along lineages Proc Natl Acad Sci USA 2004 101 12957 12962 10.1073/pnas.0402177101 15326304 PMC516501

[B60] Chennubhotla C Rader AJ Yang LW Bahar I Elastic network models for understanding biomolecular machinery: from enzymes to supramolecular assemblies Phys Biol 2005 2 S173 S180 10.1088/1478-3975/2/4/S12 16280623

[B61] Atilgan AR Durrel SR Jernigan RL Demirel MC Keskin O Bahar I Anisotropy of fluctuation dynamics of proteins with an elastic network model Biophys J 2001 80 505 515 10.1016/S0006-3495(01)76033-X 11159421 PMC1301252

[B62] Enright AJ Van Dongen S Ouzounis CA An efficient algorithm for large-scale detection of protein families Nucleic Acids Res 2002 30 1575 1584 10.1093/nar/30.7.1575 11917018 PMC101833

[B63] Vlasblom J Wodak SJ Markov clustering versus affinity propagation for the partitioning of protein interaction graphs BMC Bioinformatics 2009 10 99 10.1186/1471-2105-10-99 19331680 PMC2682798

[B64] Brohee S van Helden J Evaluation of clustering algorithms for protein-protein interaction networks BMC Bioinformatics 2006 7 488 10.1186/1471-2105-7-488 17087821 PMC1637120

